# Percutaneous Microwave Ablation of Histologically Proven T1 Renal Cell Carcinoma

**DOI:** 10.1007/s00270-020-02423-7

**Published:** 2020-02-12

**Authors:** B. M. Aarts, W. Prevoo, M. A. J. Meier, A. Bex, R. G. H. Beets-Tan, E. G. Klompenhouwer, F. M. Gómez

**Affiliations:** 1 Department of Radiology, The Netherlands Cancer Institute, Plesmanlaan 121, 1066 CX Amsterdam, The Netherlands; 2 GROW School for Oncology and Developmental Biology, Maastricht University Medical Centre, P.O. Box 5800, 6202 AZ Maastricht, The Netherlands; 3 Department of Radiology, OLVG, Oosterpark 9, 1091 AC Amsterdam, The Netherlands; 4 Department of Radiology, Isala Klinieken, Dokter van Heesweg 2, 8025 AB Zwolle, The Netherlands; 5 Department of Urology, The Netherlands Cancer Institute, Plesmanlaan 121, 1066 CX Amsterdam, The Netherlands; 6 Specialist Centre for Kidney Cancer, Royal Free London NHS Foundation Trust and UCL Division of Surgery and Interventional Science, Pond Street, London, NW3 2QG UK; 7 Department of Interventional Radiology, Hospital Clinic Universitari de Barcelona, Carrer de Villarroel 170, 08036 Barcelona, Spain

**Keywords:** Percutaneous thermal ablation, Microwave ablation, Renal cell carcinoma, Kidney

## Abstract

**Objectives:**

To assess the safety and efficacy of percutaneous microwave ablation (MWA) of histologically proven T1 renal cell carcinoma (RCC).

**Methods:**

We analysed patients with a histologically proven RCC (≤ 7 cm) treated by MWA from April 2012–April 2018. Primary and secondary efficacy, local tumour recurrence (LTR), morbidity and mortality were reported. Efficacy was defined as no residual tumour enhancement on follow-up imaging 1 month after the first ablation (primary efficacy) and after re-ablation(s) for residual disease (secondary efficacy). Adverse events (AE) were registered by the Clavien–Dindo classification and the common terminology criteria for AE. Univariable and multivariable logistic regression analyses were performed to investigate a relation among pre-treatment factors incomplete ablation and complications.

**Results:**

In 100 patients, a total of 108 RCCs (85 T1a and 23 T1b) were treated by MWA. Median size was 3.2 cm (IQR 2.4–4.0). Primary efficacy was 89% (95%CI 0.81–0.94) for T1a lesions and 52% (95%CI 0.31–0.73) for T1b lesions (*p* < 0.001). Fifteen lesions (7 T1a) were re-ablated for residual disease by MWA in one (*n* = 13) and two (*n* = 2, both T1b) sessions resulting in secondary efficacy rates of 99% (T1a) and 95% (T1b, *p* = 0.352). LTR occurred in four tumours (2 T1a, 2 T1b) after 10–60 months. Six (4%) AEs grade > 3–5 were observed (2 T1a, 4 T1b, *p* = 0.045). Multivariable analysis showed that mR.E.N.A.L. nephrometry was independently associated with incomplete ablation (*p* = 0.012).

**Conclusion:**

Microwave ablation is safe and effective for T1a and T1b RCC lesions with a significantly lower primary efficacy for T1b lesions.

## Introduction

Renal cell carcinoma (RCC) accounts for 3% of all cancers worldwide [30]. According to the European Association of Urology (EAU) and the National Comprehensive Cancer Network (NCCN) RCC guidelines, partial nephrectomy (PN) is the gold standard for T1a RCC. Percutaneous tumour ablation is reserved for co-morbid patients and patients not eligible for surgery [21, 25]. Although reports show higher local control after PN compared to ablative therapies, similar cancer-specific survival is obtained with less renal function decline for radiofrequency ablation (RFA) and cryoablation (CA) [32].

Details of the different ablation modalities, RFA, MWA and CA, are extensively described in the literature. To summarize, MWA, compared to RFA, achieves higher temperatures in a shorter time less influenced by the heat sink effect. As a result, a fast and large ablation zone with a similar applicator as RFA is achieved. [4, 20] With RFA and MWA, the evolvement of the ablation zone during the procedure is less visible compared to CA [16]. Reports about the efficacy and safety of large cohorts of MWA remain limited, especially for T1b tumours [9, 10].

In this retrospective cohort study, we report the outcomes of patients with a histologically proven RCC treated by means of MWA in a tertiary reference centre. The purpose of this study was to evaluate the safety, efficacy and factors influencing outcome of MWA in T1a and T1b RCC.

## Patients and Methods

### Study Population

The institutional review board of our hospital approved this retrospective study (IRBd18059). The data of all MWAs of our institute were requested through our institutes data desk, and consent of all patients was checked. We included patients treated by MWA for a histologically proven T1 RCC between April 2012 and April 2018. Patients were excluded when prior therapy (chemotherapy, surgical resection or a different ablation modality) for RCC was administrated.

### MWA Procedure

All patients were first discussed in a multidisciplinary tumour board, consisting of urologists, radiologists and medical oncologists to decide patients eligibility for MWA. The MW procedures were performed computed tomography (CT) guided (CT Somatom Sensation Open, Siemens^®^, Munchen, Germany). Patients were treated with two different MW systems (2012–2014: Evident^®^ MW system (Covidien^®^, Dublin, Ireland), 2014–2018: Emprint^®^ MW system (Medtronic^®^, Dublin, Ireland)). Dissection was performed with 5% glucose solution plus 10% iodinated contrast, CO2 and room air for tumours adjacent to vulnerable structures. Ureteric perfusion with cooled saline was used for tumours close to the collection system and the proximal ureter. Antenna placement was performed with CT fluoroscopy, and optimal position was verified by CT before start of the ablation. After antenna placement, biopsy was performed. In principle, a power of 100 W was used for 2–10 min according to tumour size. A margin of 5–10 mm was attempted to achieve complete ablation. Fluoroscopic CT check was performed to monitor the procedure. In larger tumours, the ablation was repeated with different antenna positions to achieve a complete ablation zone. With the Evident^®^ MW system, multiple antennas were placed in the tumour according to physicians choice.

### Follow-Up

An institutional follow-up scheme of multiphase CT scans after 1, 3, 6, 9 and 12 months was executed. Patients with a diminished renal function were followed by (non-)contrast enhanced magnetic resonance imaging (Achieva or Ingenia, Philips Healthcare^®^, Best, the Netherlands). According to agreement with our in-house urologists, follow-up for the first year was performed by the IR at our outpatient clinics. When no recurrence appeared, patients were sent back to the referring urologist. A follow-up scheme of 1 multiphase CT scan a year for 5 years was advised to the referring urologist.

### Data Collection and Statistical Analysis

Tumour characteristics were scored according to the R.E.N.A.L. nephrometry score (radius, exophytic/endophytic, nearness to collecting system or sinus and location relative to polar lines) and modified (m)R.E.N.A.L. nephrometry score, as published previously [26]. Adverse events (AEs) during ablation were registered by the common terminology criteria for adverse events (CTCAE), and post-ablation AEs were registered following the Clavien–Dindo classification. Primary efficacy was defined as no residual tumour enhancement visible at post-contrast CT or MRI 1 month post-ablation. Secondary efficacy was described as the percentage of tumours successfully treated for residual disease by repeated MWA(s) [1]. Patients treated with another secondary treatment modality (i.e. PN, radical nephrectomy (RN), RFA or CA) were excluded for the secondary efficacy. Local tumour recurrence (LTR) was defined when new enhancement within a successfully treated ablation zone occurred during the follow-up.

Continuous variables are shown as median and interquartile ranges (IQR) and categorical data as numbers and percentages. To test differences between categories, the Chi-square or Fisher exact test was used and for nonparametric continuous variables the Mann–Witney test. To analyse the relationship between pre-treatment factors with incomplete ablations and the occurrence of complications, a logistic regression analysis was performed. Results are presented as odds ratio (OR), 95% confidence interval (CI) and significance levels. For the multivariable logistic regression analysis, only significant variables from the univariable analysis were included. Significance levels of *p* < 0.05 were used. Analyses were performed using Statistical Package for the Social Sciences (SPSS, version 25, Chicago, IL).

## Results

### Patient and Tumour Characteristics

Between April 2012—April 2018, 226 patients underwent a MWA for their renal masses. One hundred and twenty-six patients were excluded because of non-diagnostic biopsy (*n* = 55), benign biopsy (*n* = 30), treatment for recurrence disease after prior treatment (*n* = 12), metastatic disease (*n* = 23), T3 disease (*n* = 2), tumour debulking (*n* = 2), prior chemotherapy for tumour reduction (*n* = 1) and no follow-up imaging available after the MWA (*n* = 1). One hundred patients with 108 histologically proven RCCs were included in this analysis; patient and tumour characteristics are shown in Table 1.

**Table 1:** Pre-treatment characteristics

	All (*n* = 108)	T1a (*n* = 85)	T1b (*n* = 23)	*p* value
Number of patients	100	77	23	
Median age at treatment, IQR	71 (63–77)	69 (63–76)	74 (63–77)	0.285^a^
Male	59 (59%)	48 (62%)	11 (48%)	0.235^b^
History				
Cardiovascular	30 (30%)	21 (25%)	9 (39%)	0.306^b^
Oncological	38 (38%)	29 (38%)	9 (39%)	0.899^b^
Urological	22 (22%)	15 (19%)	7 (30%)	0.389^b^
Median size, IQR	3.2 (2.4–4.0)	2.8 (2.2–3.5)	4.5 (4.3–5.0)	0.000^a^
Laterally				0.357^b^
Right	56 (52%)	42 (49%)	14 (61%)	
Left	52 (48%)	43 (51%)	9 (39%)	
Aetiology tumour				0.096^b^
Clear cell	68 (63%)	51 (60%)	17 (74%)	
Papillary	22 (20%)	21 (25%)	1 (4%)	
*Type I*	*14*	*14*	*0*	
*Type II*	*4*	*3*	*1*	
Chromophobe	4 (4%)	4 (5%)	0	
Eosinophilic	1 (1%)	0	1 (4%)	
Undefined renal cell carcinoma	13 (12%)	9 (11%)	4 (17%)	
Fuhrman grade				0.501^b^
1	23 (21%)	17 (20%)	6 (26%)	
2	32 (30%)	23 (27%)	9 (39%)	
3	6 (6%)	5 (6%)	1 (4%)	
Undefined/not possible	47 (44%)	40 (47%)	7 (31%)	
Location				0.175^b^
Exophytic	61 (57%)	44 (52%)	17 (74%)	
< 50% exophytic	11 (10%)	10 (12%)	1 (4%)	
Endophytic	36 (33%)	31 (36%)	5 (22%)	
Anterior	26 (24%)	23 (27%)	3 (13%)	0.076^2^
Posterior	62 (57%)	44 (52%)	18 (78%)	
Mid	20 (19%)	18 (21%)	2 (9%)	
Lower pole	31 (29%)	25 (29%)	6 (26%)	0.874^b^
Upper pole	35 (32%)	28 (33%)	10 (43%)	
Inter pole	42 (39%)	32 (38%)	7 (30%)	
Distance to collecting system				**0.004**^b^
> 7 mm	66 (61%)	58 (68%)	8 (35%)	
4–7 mm	11 (10%)	9 (11%)	2 (9%)	
< 4 mm	31 (29%)	18 (21%)	13 (57%)	
Distance to polar lines				0.155^2^
Entirely above/below	62 (57%)	52 (61%)	10 (43%)	
Lesion crosses 1 polar line	27 (25%)	21 (25%)	6 (22%)	
> 50% of mass across polar line	19 (18%)	12 (14%)	7 (35%)	
R.E.N.A.L. nephrometry score	6 (4–8)	5 (4–7)	7 (6–9)	**0.000**^**a**^
Low (4–6)	68 (63%)	60 (71%)	8 (35%)	**0.002**^**b**^
Intermediate (7–9)	28 (26%)	17 (20%)	12 (52%)	**0.007**^b^
High (10–12)	12 (11%)	8 (9%)	4 (18%)	0.280^b^
m.R.E.N.A.L. nephrometry score	6 (5–9)	5 (4–8)	8 (7–10)	**0.000**^a^
Low (4–6)	59 (55%)	55 (65%)	4 (17%)	**0.000**^b^
Intermediate (7–9)	29 (27%)	19 (23%)	10 (44%)	0.062^b^
High (10–12)	20 (19%)	11 (13%)	9 (40%)	**0.004**^a^

^a^Mann–Witney test ^b^Chi-square, *IQR* interquartile range

Bold values indicate the significance level of *p* < 0.05

### Primary and Secondary Efficacy

A total of 125 MW ablations were performed in 108 tumours. The Evident^®^ MW system was used in 14 MWAs with the use of multiple antennas (2–3) in 10 tumours. The Emprint^®^ MW system was used in the other 111 MWAs. Dissection was used in 37% of the procedures with five ureter perfusions. Patients were mostly placed in the CT scanner in a prone position (75%), under epidural (98%) or general (2%) anaesthesia.

**Fig. 1:**
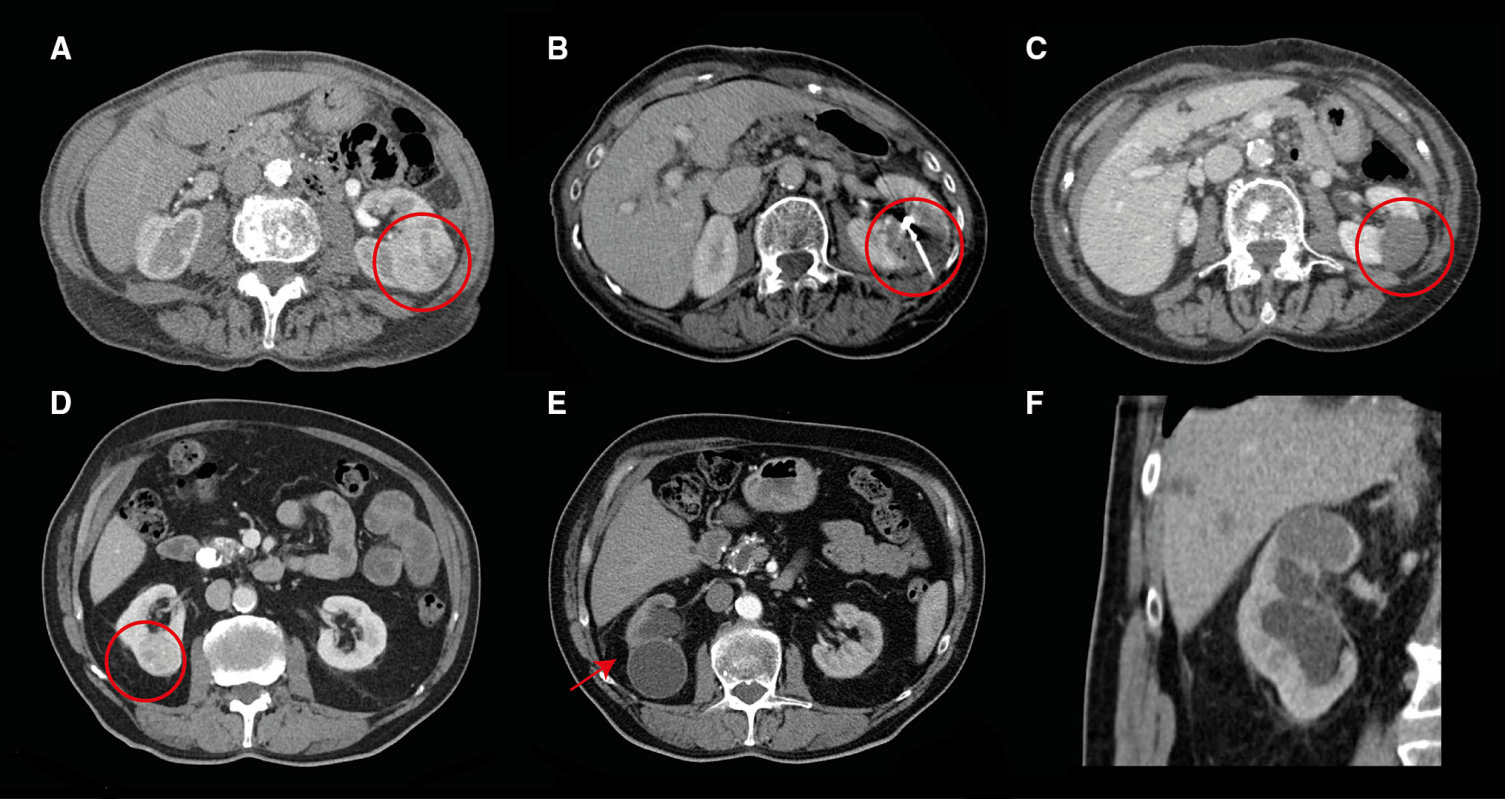
**A**–**C** Microwave ablation (MWA) of a T1b tumour (**A**) before MWA (**B**) during MWA (**C**) 1 year after MWA: complete ablation. **D**–**F** endophytic T1a lesion with a close relation to the collecting system (**D**). **E** + **F** 9 months after complete ablation, hydronephrosis of the kidney visible due to an urinary tract stenosis that occurred 3 months after the MWA (kidney function from 45  to 19 ml/min/1.73m2) (NB this patient is familiar with liver cysts)

Primary efficacy was achieved in 88 lesions (81%) (Table 2, Fig. 1A–C). T1a lesions had a significantly higher primary efficacy (89%; CI 0.81–0.94) compared to T1b lesions (52%; CI 0.31–0.73) (*p* < 0.001). Fifteen tumours (53% T1b) received a second MWA and two T1b tumours a third MWA. Secondary efficacy of MWA was reached in 97% (101/103) (all tumours), 99% (82/83) (T1a) and 95% (19/20) (T1b, *p* = 0.352). Five tumours (5/20, 2 T1a and 3T1b) were not re-treated by MWA and excluded for the secondary efficacy, but were all successfully treated by means of surgery (PN; T1a) and other ablative techniques (RFA (1 T1a tumour (1 × re-RFA) and 1 T1b tumour (3x re-RFA)), CA (*n* = 1; T1b)) and no treatment (patients choice). In five lesions, the second MWA was incomplete (1 T1a, 4 T1b) and were successfully treated with MWA (*n* = 2). In three lesions, another ablative modality was used (CA (*n* = 2) and RFA (*n* = 1)).

**Table 2:** Efficacy results for patients treated for a T1a and T1b RCC lesion

	All	T1a	T1b	*p* value
Primary efficacy	88/108 (81%)	76/85 (89%)	12/23 (52%)	**< 0.001**
Remnant	20/108 (19%)	9/85 (11%)	11/23 (48%)	**< 0.001**
Secondary efficacy	101/103 (98%)	82/83 (99%)	19/20 (95%)	0.352
Recurrence	3/105 (3%)	2/83 (2%)	2/22 (9%)	0.193

Bold values indicate the significance level of *p* < 0.05

### Adverse Events

A total of 24 (19%) AEs were observed after the 125 MW procedures including six major AEs (4%) with a significantly higher number of major AEs in T1b tumours (2 T1a 2% and 4T1b 13%, *p* = 0.045) (Table 3). There was no significant difference in the occurrence of all complications and T stage (18 T1a 20% and 6 T1b 18%, *p* = 1.000). One major AE consisted of an active bleeding on the control scan during the MWA, which was successfully coiled embolized (CTCAE grade 3). One patient died 13 days after the ablation due to cardiac and renal failure (Clavien–Dindo grade 5). In two patients, a urinary tract stenosis arose after the MWA (Clavien–Dindo 4a) after 1 and 2 months resulting in a non-functional kidney (see Fig. 1D–F). In three patients, a urinary tract stenosis occurred 1, 2 and 5 months after the MWA without loss of renal function (Clavien–Dindo 1). All five lesions were endophytic T1a lesions, with a close relation to the collection system (4 lesions < 4 mm, 1 lesions 4–7 mm). Cooling of the urinary tract system was performed in one lesion. One patient with macroscopic haematuria required transfusion with packed red blood cells and antibiotics (both grade 3). Nineteen (15%) minor AEs occurred (Clavien–Dindo grade 1 + 2).

**Table 3:** Adverse events during and post-ablation

Adverse events	Grade^a^	*n*	T stage
During ablation			
Tumour bleeding required embolization	3	1	T1b
Post-ablation			
Dead within 30 days	5	1	T1b
Urinary tract stenosis with renal function loss	4a	2	T1a
Ureteral blood loss required endoscopic intervention	3	2	T1b
Infection treated with antibiotics	2	2	T1a,b
Urinary tract stenosis *(without renal function loss)*	1	3	T1a
Self-limiting (peri-renal or liver) bleeding	1	4	T1a,b
Pneumothorax	1	3	T1a
Sensibility loss of skin	1	1	T1a
Pain	1	2	T1a
Nausea	1	2	T1a,b
Skin burn^b^	1	1	T1a

^a^According to the common terminology criteria for adverse events during ablation and according to the Clavien–Dindo Classification post-ablation

^b^The skin burn occurred at the antenna insertion and was successfully treated with silver sulfadiazine cream

### Factors Influencing Outcome

R.E.N.A.L. nephrometry score (OR 1.56, *p* = 0.001), mR.E.N.A.L. nephrometry score (OR 1.58, *p* = 0.000) and tumour aetiology (clear cell vs non-clear cell OR 0.11, *p* = 0.034) were significantly associated with an incomplete ablation; however, only the mR.E.N.A.L. nephrometry score remained significantly associated in the multivariable regression analysis (OR = 3.854 *p* = 0.012) (Table 4). Of the mR.E.N.A.L nephrometry score, size (> 3 and > 4), nearness to the collecting system (< 4 mm) and distance to the polar lines (score 3) were significantly associated with an incomplete ablation.

**Table 4:** Pretreatment factors and their association with an incomplete ablation and the occurrence of complications

Variable	Test	Univariable^a^			Multivariable^b^		
		OR	95% CI	*p* value	OR	95% CI	*p* value
Incomplete ablation							
Tumour aetiology	Clear cell versus non-clear cell	**0.107**	**0.01–0.85**	**0.034**	0.077	0.04–1.3	0.077
Fuhrman grade^c^	Grade 1 versus grade 2 + 3	1.286	0.4–4.3	0.688	–	–	–
	Grade 1 versus unknown	0.429	0.1–1.7	0.221	–	–	–
Age	Continue	1.005	0.96–1.06	0.833	–	–	–
Gender	Male versus Female	0.770	0.3–2.1	0.600	–	–	–
History cardiovascular	Yes versus no	0.714	0.2–2.2	0.551	–	–	–
History oncological	Yes versus no	0.492	0.2–1.3	0.157	–	–	–
History urological	Yes versus no	0.882	0.3–2.7	0.882	–	–	–
Tumour location	Left versus right	1.500	0.6–4.0	0.421	–	–	–
	Anterior versus posterior	0.391	0.08–1.9	0.242	–	–	–
	Anterior versus inter	0.720	0.2–2.4	0.589	–	–	–
System	Emprint versus evident	0.342	0.1–1.2	0.086	–	–	–
R.E.N.A.L. nephrometry score	Continue	**1.559**	**1.2–2.0**	**0.001**	0.356	0.1–1.1	0.069
mR.E.N.A.L. nephrometry score	Continue	**1.577**	**1.2–2.0**	**0.000**	**3.854**	**1.3–11**	**0.012**
Complications							
Age	Continue	0.994	0.95–1.0	0.772	–	–	–
Gender	Male versus female	1.063	0.4–2.6	0.895	–	–	–
History cardiovascular	Yes versus no	0.729	0.3–2.0	0.542	–	–	–
History oncological	Yes versus no	0.941	0.4–2.3	0.895	–	–	–
History urological	Yes versus no	1.234	0.4–3.6	0.704	–	–	–
Tumour location	Left versus right	1.346	0.6–3.3	0.517	–	–	–
	Anterior versus posterior	0.682	0.3–2.0	0.521	–	–	–
	Anterior versus inter	0.692	0.2–2.7	0.598	–	–	–
Dissection	Yes versus no	1.091	0.4–2.8	0.856	–	–	–
System	Emprint versus evident	1.314	0.3–5.2	0.697	–	–	–
R.E.N.A.L. nephrometry score	Continue	**1.308**	**1.1–1.6**	**0.013**	–	–	–
mR.E.N.A.L. nephrometry score	Continue	**1.226**	**1.1–1.5**	**0.016**	–	–	–

Bold values indicate the significance level of *p* < 0.05

^a^In the univariable analysis, all variables were analysed to determine the relation of (m)R.E.N.A.L. nephrometry score and their components with incomplete ablation and complication

^b^In het multivariable analysis, only the significant variables from the univariable factors were used

^c^Fuhrman grade is a nuclear grading system of clear cell RCC that evaluated the nuclear size, shape and nucleolar prominence

Univariable analysis showed that only the R.E.N.A.L. nephrometry score (OR 1.308, *p* = 0.013) and mR.E.N.AL. nephrometry score (OR 1.577, *p* = 0.016) were associated with the occurrence of complications. Of the (m)RENAL nephrometry score, only the nearness to the collecting system (< 4 mm) was significantly associated with the occurrence of complications.

### Follow-Up

Median follow-up time was 19 months (IQR 12–35 months; min–max 0–78 months, 90 patients (90%) 1-year follow-up available). During this period, four (4%) tumours showed LTR (2 T1a and 2 T1b tumours) after 10, 13, 26 and 60 months. One recurrence was successfully treated by MWA and two recurrences by another modality (CA). One of the recurrences has not been treated yet. One patient treated by MWA for bilateral chromophobe RCC tumours developed a new lesion in the same kidney for which he underwent active surveillance. Five patients (4 T1a tumours, 1 T1b tumours) developed metastases of which two were histologically proven RCC. During follow-up, no patient died of RCC.

## Discussion

Percutaneous ablation is considered as treatment option in co-morbid patients with a T1 RCC tumour not eligible for PN [21, 25]. RFA and CA are widely applied and established ablation techniques for RCC that are included in the guidelines contrary to MWA which has still limited supportive data [21, 25, 27].

In this study, we show the result of 108 RCCs treated with MWA. There was a significantly higher primary efficacy for T1a tumours (89%) compared to T1b tumours (52%). In the 125 performed MWAs, 19% AEs were observed, mostly low grade (15%), with a significantly higher number of major AEs in T1b tumours (13% T1b vs 2% T1a, *p* = 0.045). The R.E.N.A.L. score and mR.E.N.A.L. nephrometry score were related to incomplete first ablation and the occurrence of complications. The factors, size (> 3 cm and > 4 cm), nearness to the collecting system (< 4 mm) and distance to the polar lines (score 3) of the mR.E.N.A.L score were associated with incomplete ablation and nearness to the collecting system (< 4 mm) for the occurrence of complications.

For all primary MWAs, a primary efficacy of 81% was observed with a primary efficacy of 89% for T1a tumours. After repeated MWA(s), a secondary efficacy of 99% was reached for T1a tumours. In the literature, primary efficacy rates of MWA from 84.6 to 100% are reported [8, 11, 14, 22, 29]. Therefore, the primary and secondary efficacy of T1a tumours underpins the existing evidence supporting MWA for the treatment of T1a RCC lesions.

The primary efficacy of T1b tumours (52%) was significantly lower compared to T1a tumours (*p* < 0.001). In 48% of the T1b lesions, a second or third ablation was performed to achieve a complete tumour ablation resulting in a secondary efficacy of 95%. In the literature, lower efficacy rates of percutaneous ablation are described for tumours over 4 cm [29] which is in line with our findings. We show that repeated MW ablations can achieve high efficacy rates even in large tumours. Besides the tumour size, our cohort contained difficult tumours with a close relation to the collecting system. Reports of percutaneous ablation of T1b RCCs by MWA are rare, and series are small [3, 9]. Primary efficacy rates between 75 and 100% were reported in 12 and 7 T1b tumours, respectively [12, 33]. In the literature, CA is more commonly used as ablation technique for T1b lesions with primary efficacy rates ranging from 76 to 97.2% [2, 5, 13, 15]. Our results show that MWA can also be used and chosen as a treatment modality in T1b tumours with consideration of a second ablation. Future cost-effectiveness studies have to show the exact place of the difference ablation techniques for RCC.

The overall AE rate of 19% was relatively high compared to previous percutaneous ablation and surgical studies [9, 32]. However, most AEs were low grade (15%) with minimal consequences for the patient. In our series, we found a significant difference between T1a and T1b tumour for the occurrence of major AEs. Five patients had a stenosis of the urinary tract, 1–5 months after MWA that resulted in renal function loss in two patients. Nearness to the collecting system was significantly associated with incomplete ablation and complications. Therefore, we suggest caution during MWA for lesions close to the collecting system. In the literature, 13 cases of injury to the urinary tract system after MWA are reported [7, 11, 19, 23, 31, 33]. Klapperich et al. observed six asymptomatic urinomas that resulted in renal cortex volume loss in three patients [19]. Preclinical work on a histologically level shows damage of MWA to the collecting system by direct puncture of the collecting system and heating of the urine during the ablation [24, 28]. Damage to the gastrointestinal tract was reported by others, but not observed in the current study [16, 18].

Multivariable analysis showed an independent association of the mR.E.N.A.L. nephrometry score for an incomplete first ablation. In addition, univariable analysis showed a relation of R.E.N.A.L. and m.R.E.N.A.L. nephrometry score with the occurrence of complications. These results are in line with Ierardi et al. that also reported an association of the (m)R.E.N.A.L. nephrometry score with incomplete ablation and complications after MWA [17] and Camacho et al. that first described this association after RFA and CA [6]. On the contrary, Klapperich et al. only found an association between local recurrence after MWA and tumour histology characteristic, and not between the R.E.N.A.L. score and local recurrence [19]. Shakeri et al. observed a significantly higher median tumour size in lesions that required a second MWA, but no association with tumour location and R.E.N.A.L score [29]. Also, Wells et al. did not find an association with the RENAL score and treatment outcome [33]. These reports are all opposite to our findings which may suggest that MWA is not as straight forward as previously reported.

The LTR in this study was 4% over a 108 histologically proven RCCs. In the literature, LTR ranges from 0 to 17% [7, 12, 29], but most papers included every renal lesions without excluding non-diagnostic or benign lesions whereby efficacy and recurrence rates might be overestimated [23, 31, 33]. Surprisingly, the time to LTR was long in this current cohort which could be explained by the difficulties in detecting of recurrences in hypo-vascular tumours and slow growth rates of low-grade tumours.

Limitations of this study include a single-centre retrospective study whereby details of the MW procedure were not complete with the lack of in-house long-term follow-up (> 1 year) in some patients. Ideally, we would describe a larger cohort, but the peri-operative biopsy strategy resulted in some non-diagnostic and benign lesions.

In conclusion, primary efficacy of MWA in T1a tumours was significantly higher compared to T1b tumours. Repeated ablation was necessary in 19% achieving efficacy rates of 95% (T1a) and 99% (T1b). Low-grade AEs were seen after MWA whereby close monitoring of the urinary tract is recommended following ablation of tumours adjacent to the urinary tract. Incomplete ablation was more often seen in lesions with a larger size with a close relation to the collecting system and the polar lines accompanied expressed in a higher mR.E.N.A.L. nephrometry score. Prospective data have to determine the exact position of MWA for the treatment of RCC.

## Abbreviations

RCC: Renal cell carcinoma
EAU: European association of urology
NCCN: National comprehensive cancer network
PN: Partial nephrectomy
RFA: Radiofrequency ablation
CA: Cryoablation
MWA: Microwave ablation
R.E.N.A.L.: Radius, exophytic/endophytic, nearness to collecting system or sinus and location relative to polar lines
mR.E.N.A.L.: Modified R.E.N.A.L
AE: Adverse event
CTCAE: Common terminology criteria for adverse events
LTR: Local tumour recurrence
CT: Computed tomography
HR: Hazard ratio
IQR: Interquartile range
RN: Radical nephrectomy
ROC: Receiver operating characteristics
AUCs: Area under the curve
